# The analysis of respiration-induced pancreatic tumor motion based on reference measurement

**DOI:** 10.1186/1748-717X-9-192

**Published:** 2014-08-30

**Authors:** Lukas Knybel, Jakub Cvek, Bretislav Otahal, Tomas Jonszta, Lukas Molenda, Daniel Czerny, Eva Skacelikova, Marian Rybar, Pavel Dvorak, David Feltl

**Affiliations:** Department of Oncology, University Hospital Ostrava, 17. listopadu 1790, 708 52 Ostrava, Czech Republic; Department of Radiology, University Hospital Ostrava, Ostrava, Czech Republic; Medical Physics, The London Clinic, London, UK

**Keywords:** Pancreatic cancer, Tumor motion, Internal target volume

## Abstract

**Background:**

To evaluate pancreatic tumor motion and its dynamics during respiration.

**Methods and materials:**

This retrospective study includes 20 patients with unresectable pancreatic cancer who were treated with stereotactic ablative radiotherapy. An online respiratory tumor tracking system was used. Periodical maximum and minimum tumor positions with respiration in superior-inferior (SI), latero-lateral (LL), and anterior-posterior (AP) directions were collected for tumor motion evaluation. The predictability of tumor motion in each axis, based on reference measurement, was analyzed.

**Results:**

The use of a 20-mm and 5-mm constant margins for SI and LL/AP directions, avoids target underdosage, without the need for reference measurement. Pearson’s correlation coefficient indicated only a modest correlation between reference and subsequent measurements in the SI direction (r = 0.50) and no correlation in LL (r = 0.17) and AP (r = 0.35) directions. When margins based on the reference measurement of respiratory tumor motion are used, then 30% of patients have a risk zone of underdosage >3 mm (in average). ITV (internal target volume) optimization based on the reference measurement is possible, but allows only modest margin reduction (approximately from 20 mm to 16-17 mm) in SI direction and no reduction in AP and LL directions.

**Conclusion:**

Our results support the use of 20-mm margin in the SI direction and 5-mm margins in the LL and AP directions to account for respiratory motion without reference measurement. Single measurement of tumor motion allows only modest margin reduction. Further margin reduction is only possible when there is on-line tumor motion control according to internal markers.

## Background

Pancreatic carcinoma is a leading cause of cancer-related mortality. Although surgery is the standard treatment of pancreatic cancer, only 20% of patients are diagnosed with resectable disease [1]. The outcomes after chemoradiation for unresectable pancreatic cancer are poor, mainly because commonly used doses are not lethal for adenocarcinoma. The irradiated volume is correlated with significant gastrointestinal toxicity [2]. Moreover, dose escalation is not possible without exceeding normal tissue dose constraints while including regional lymph nodes [3]. The reduction of the conformal fields to include only the gross tumor volume (GTV) plus margins to account for microscopic disease (clinical target volume, CTV), tumor motion (internal target volume, ITV) and treatment setup, (planning target volume, PTV) results in better tolerance [4–6]. The recently published American-French Consensus proposes that PTV includes the GTV, with a shaped aperture margin of 15 to 20 mm in LL and AP directions and a margin of 20 to 30 mm in the SI direction, to take into account microscopic spreading, respiratory movements and set-up margin [7].

Performance of four-dimensional computed tomography (4D-CT) simulation with the creation of an ITV might reduce the margins used to account for respiration [8]. Controversy exists regarding whether one measurement of tumor motion can predict future movement [9, 10]. Excessive volume reduction can lead to excursions outside the designated PTV margin, resulting in underdosage of the target. On the other hand, larger margins will lead to unnecessary irradiation of organs at risk (OARs).

In this study we analyzed pancreatic tumor motion in detail during a period of >3 h for each case and evaluated the application of several compensatory mechanisms to avoid systemic errors.

## Materials and methods

This retrospective study includes 20 patients with unresectable pancreatic cancer (11 women, 9 men) who were treated between December 2011 and August 2012. We used the CyberKnife® Robotic Radiosurgery System and Synchrony respiratory tracking system (Accuray Inc., Sunnyvale, CA). Plans were designed to cover 95% of PTV (CTV based on CT/MRI registration during relaxed exhale + 3-mm safety margin) with the prescribed dose. A total of 20 tumors were treated with three fractions of 10 Gy every other day. In total, 60 fractions were analyzed. Four gold markers (fiducials) were implanted percutaneously under the CT control (each fiducial within 30 mm from tumor center and fiducial constellation centroid within 10 mm of the tumor center). It is assumed that motion of the fiducial’s center of mass (COM) closely approximates to the motion of the tumor’s COM. Constrains for OARs were set: 10 ml < 21Gy, 5 ml < 15Gy, 700 ml < 17Gy and 0,25 ml < 18Gy for stomach, duodenum, liver and spinal cord respectively. Patients were asked to breathe normally during the irradiation.

### Tumor motion detection

The Synchrony® Respiratory Tracking System [11] records the breathing light signal from external markers on the chest and relates this signal to the X-ray coordinates of internal markers. The system calculates modelling error and an adaptive algorithm is used for online estimation of internal/external marker correlation error during the entire session (image frequency > 1 per minute). The geometrical coordinates representing position of the fiducials in SI, LL and AP directions in time are extracted from the treatment X-ray images to “log” files. This method has been proven to have high accuracy regarding the evaluation of tumor motion [12].

Reference measurement was calculated prior to treatment (in the day of planning CT) from the correlation model, based on the average value of tumor motion amplitudes within 2-5 minutes (at least 8 X-ray images to make correlation model, 5 X-ray images for confirmation of correlation model stability) or using constant upper/lower margins from American – French consensus. All CyberKnife treatments involve an initial intrafraction alignment step (checking the position of both spine structures and fiducial markers), followed by continuous intrafraction respiratory motion monitoring and determination [11]. According to the patient’s precise setup (spine alignment with error less than 1 mm), no other data corrections were needed. Before each fraction, the correlation model must have been created. The analyzed data covered 95% of the total treatment time. Over 5000 positions of COM were recorded during the respiration cycles; approximately 800 of these, representing the maximum or minimum amplitude of tumor motion (periodical expiration or inspiration peak), were used for analysis.

### Tumor motion analysis

To evaluate possible margins, under/overestimation of the tumor motion based on the reference measurement, we have derived the following formulas:


Where:

TAU - the time averaged margin underestimation,

TAO - the time averaged margin overestimation,

x_margin_ - the margin used for ITV determination (value from reference measurement or predefined value from American- French consensus),

x_j_ - the periodical tumor motion amplitude during the j-th portion of treatment,

t_j_ - the duration of the j-th portion of treatment (period with stable correlation model),

t_1_ + t_2_ + …t_n_ – the duration of the whole treatment (n is number of correlation models),

u - the number of portions of the treatment with underestimated margins,

o - the number of portions of the treatment with overestimated margins.

**Figure 1:**
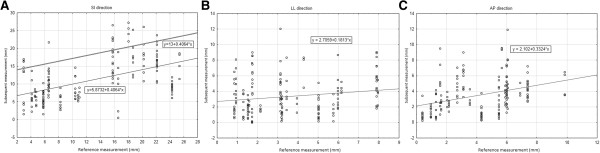
**Regression analysis with equations for SI (A), LL (B), and AP (C) directions.** Pearson’s correlation coefficient was 0.501 (p = 0.000), 0.1719 (p = 0.0225), and 0.3461 (p = 0.000) for SI, LL, and AP, respectively. Same and similar values are overlapping. 1A: The lowest line (y = 5,8732 + 0,4064*x) is the result of regression analysis. The upper line (y = 13 + 0,4064*x) is the result of TAU and TAO optimization.

We used regression analysis to evaluate whether one or more measurements of tumor motion can adequately represent the motion of pancreatic tumors and to describe intra- and interfractional variation of tumor motion. We used linear regression function for modeling of influence of the reference and subsequent measurements in the SI direction. The linear function has the form y = b_0_ + b_1*_x, where b_0_ equals a constant of the function (intercept on the vertical axis), b_1_ equals the slope of the linear function, x represents the first reference measurement and y represents subsequent measurements (Figure 1A). Our margin optimization uses the shift of the linear regression line. This approach was selected in order to lower the TAO when TAU is predefined (the same as in the American-French approach). Our new approach respects individual patient amplitudes of tumor movements caused by respiration, and enables the setting of margins of radiation to individual patients based on their reference measurement. The shift of the linear regression line along the vertical axis is made experimentally in order to find TAU nearest to the predefined value. The constant part of equation (b_0_) is changed by the shift, while the slope (which is defined by the function) remains constant. TAO is calculated from the formula afterwards.

### Statistical analysis

Statistical analysis was performed using STATISTICA 10 software (Statsoft, Tulsa, OK). We used the Test for Difference Between Two Correlation or Regression Coefficients to distinguish between males and females, and the Test for Difference Between Two Regression Coefficients to distinguish between the slope of influence of respiration amplitudes on the reference measurement.

## Results

The amplitude of COM motion during respiration varied widely among patients (range 7.3-27.3 mm in SI direction) and could vary intra-fractionally with a variation coefficient greater than 25%, for 25% of patients; moreover, the change of the tumor movement could be seen unexpectedly.

### Margins to account respiration without a reference measurement

Using a margin from the upper limit (20 mm) for the SI direction (considered in the American-French consensus) resulted in low average TAU, and only one tumor has been presented with TAU >3 mm. Unfortunately, the use of this margin caused TAO greater than 8 mm, on average (Table 1). When a margin from the lower limit (10 mm) for the SI direction was used, the risk zone of TAU was >3 mm; moreover, 8 out of 20 tumors (40%) can be missed by >3 mm (range 3.5-13 mm).

**Table 1:** **Pancreas movement in SI direction during three fractions, TAO and TAU for lower and upper limit for SI direction considered in the American-French consensus**

Superior- Inferior							
	Motion (mm)			Margin 10mm		Margin 20mm	
Patient	Max	Mean	Std	TAU	TAO	TAU	TAO
1	19.1	10.7	7.0	4.3	2.5	0.0	8.2
2	11.0	8.5	2.0	0.3	0.8	0.0	10.9
3	10.8	7.0	2.2	0.2	2.4	0.0	13.0
4	7.3	4.8	1.6	0.0	4.6	0.0	14.6
5	25.3	17.8	5.1	8.8	0.0	0.7	2.0
6	13.7	6.5	3.5	1.6	1.3	0.0	9.6
7	13.8	9.3	2.6	0.6	0.6	0.0	9.9
8	26.6	16.9	4.6	9.9	0.0	1.8	1.9
9	24.3	9.8	3.8	1.1	1.0	0.3	10.2
10	17.0	9.8	6.4	2.0	4.0	0.0	12.0
11	14.6	9.2	3.0	0.8	1.6	0.0	10.7
12	21.7	11.7	3.9	3.5	0.2	0.3	6.9
13	27.3	23.4	2.2	13.0	0.0	3.6	0.7
14	23.7	17.1	3.6	7.6	0.0	0.5	2.8
15	10.3	7.1	1.8	0.1	2.6	0.0	12.6
16	8.9	5.3	2.1	0.0	4.1	0.0	14.1
17	26.1	17.6	4.4	10.2	0.0	1.9	1.7
18	8.5	6.8	1.5	0.0	2.8	0.0	12.8
19	25.2	17.5	4.3	7.3	0.0	0.2	2.5
20	7.9	5.2	1.5	0.0	3.8	0.0	13.8
Mean (range)	17.2 (7.3-27.3)	11.1 (4.8-23.4)		3.6	1.6	0.5	8.5

Results were different for the LL and AP directions; the use of a margin close to the lower range of recommendation (5 mm) generated low TAU and TAO. When a higher limit (10 mm) was used, only a very small improvement of TAU was calculated, and TAO increased unnecessarily (Tables 2 and 3).

**Table 2:** **Pancreas movement in LL direction during three fractions, TAO and TAU for lower and upper limit for LL direction considered in the American-French consensus**

Latero-Lateral							
	Motion (mm)			Margin 5mm		Margin 10mm	
Patient	Max	Mean	Std	TAU	TAO	TAU	TAO
1	3.5	2.0	1.4	0.0	2.9	0.0	7.9
2	3.9	2.7	1.1	0.0	2.3	0.0	7.3
3	6.1	4.8	0.9	0.3	0.5	0.0	5.4
4	6.5	2.6	1.9	0.2	2.3	0.0	7.1
5	7.9	4.7	2.2	0.6	1.1	0.0	5.5
6	7.7	1.9	2.4	0.9	0.9	0.0	5.5
7	4.2	2.1	1.5	0.0	3.2	0.0	8.3
8	9.1	5.5	2.3	1.9	0.4	0.0	3.5
9	5.0	1.9	1.5	0.0	2.7	0.0	7.7
10	8.7	4.0	2.1	0.2	2.2	0.0	7.0
11	3.2	1.8	0.9	0.0	2.9	0.0	7.9
12	8.6	6.0	2.0	2.2	0.1	0.0	2.9
13	8.3	6.7	1.8	1.8	0.3	0.0	3.5
14	12.1	4.8	2.4	0.9	1.1	0.0	5.1
15	2.2	1.8	0.3	0.0	3.2	0.0	8.2
16	2.9	1.8	0.9	0.0	3.0	0.0	8.0
17	8.1	4.3	2.2	0.8	0.9	0.0	5.1
18	2.8	2.0	0.6	0.0	2.9	0.0	7.9
19	5.7	2.0	1.9	0.2	2.2	0.0	7.0
20	8.3	4.2	2.2	1.1	1.0	0.0	4.8
Mean (range)	6.2 (6.5-8.3)	3.4 (2.6-6.7)		0.5	1.8	0.0	6.3

**Table 3:** **Pancreas movement in AP direction during three fractions, TAO and TAU for lower and upper limit for AP direction considered in the American-French consensus**

Anterior-Posterior							
	Motion (mm)			Margin 5mm		Margin 10mm	
Patient	Max	Mean	Std	TAU	TAO	TAU	TAO
1	6.0	3.0	1.6	0.2	1.7	0.0	6.4
2	6.9	4.8	1.6	0.7	0.5	0.0	4.8
3	4.3	3.1	0.8	0.0	2.1	0.0	7.1
4	5.9	2.9	1.9	0.3	2.1	0.0	6.9
5	9.8	5.9	2.6	1.5	0.1	0.0	3.7
6	9.5	4.7	2.9	0.4	0.1	0.0	3.2
7	4.9	3.3	1.2	0.0	2.1	0.0	7.1
8	7.4	4.9	1.5	0.9	0.3	0.0	4.5
9	4.3	1.3	1.1	0.0	3.4	0.0	8.4
10	6.0	4.3	1.5	0.4	1.3	0.0	5.9
11	2.4	1.4	0.6	0.0	3.6	0.0	8.6
12	9.0	5.6	1.8	1.3	0.3	0.0	4.0
13	9.5	8.2	1.2	2.8	0.0	0.0	2.2
14	7.4	4.6	1.4	0.4	0.9	0.0	5.5
15	2.8	2.2	0.4	0.0	2.7	0.0	7.7
16	3.4	0.9	0.8	0.0	4.1	0.0	9.1
17	11.9	5.6	3.0	2.7	0.4	0.5	3.2
18	3.9	2.8	0.8	0.0	2.2	0.0	7.2
19	5.9	3.8	1.2	0.3	1.0	0.0	5.7
20	3.5	2.4	0.7	0.0	2.8	0.0	7.8
Mean (range)	6.2 (5.9-9.5)	3.8 (2.9-8.2)		0.6	1.6	0.0	5.9

### Margins to account respiration with a reference measurement

Pearson’s correlation coefficient indicated a modest correlation in the SI direction (Figure 1A) and very poor correlations in the LL and AP directions (Figure 1B, C). Given these results, we could not hypothesize any way to predict margins that would account for motions in the LL and AP directions based on reference measurements.

Using margins in the SI direction based on the reference measurement of respiratory tumor motion allowed us to avoid overdosage, with the average geometrical miss better than 3 mm (Table 4). However, 30% of patients had a risk zone of underdosage that measured >3 mm (range 3.6 – 7.4 mm)

**Table 4:** **Summary table of the different concepts to determine ITV in SI direction**

Margin strategy	Constant 10mm	Constant 20mm	Reference measurement	Constant 15 or 20 mm	Constant 15 or 20 mm- man	Constant 15 or 20 mm- woman	Regression analysis all, y = 13 + 0.4064•x	Regression analysis- man y = 8.5 + 0.7015•x	Regression anylysis- woman y = 17 + 0.1804•x
TAU (mm)	3.6	0.5	2.3	0.5	0.3	0.7	0.5	0.3	0.7
TAU > 3mm (%)	40%	5%	30%	5%	0%	9%	5%	0%	9%
TAU > 1mm (%)	55%	15%	50%	20%	11%	27%	15%	11%	18%
TAO (mm)	1.6	8.5	2.7	5.6	6.2	5.1	6.7	5.6	7.4
TAO > 3mm (%)	20%	70%	25%	65%	78%	55%	85%	67%	73%
TAO > 1mm (%)	55%	95%	50%	95%	100%	91%	95%	100%	91%
Margin- average (mm)	10	20	12.1	17	16.7	17.3	17.9	15.8	19.4

Closer evaluation of the results clearly revealed the segregation of our patients into two groups, one with movement more than 15 mm, and the second with movement less than 15 mm in the SI direction (Figure 1A). To keep TAO and TAU as low as possible for patients with both types of breathing patterns, ITV margins had to be adjusted accordingly: 15 mm for shallow breathing and 20 mm for deep breathing (Table 4). Similar segregation was not observed for LL or AP directions (Figure 1B, C).

**Figure 2:**
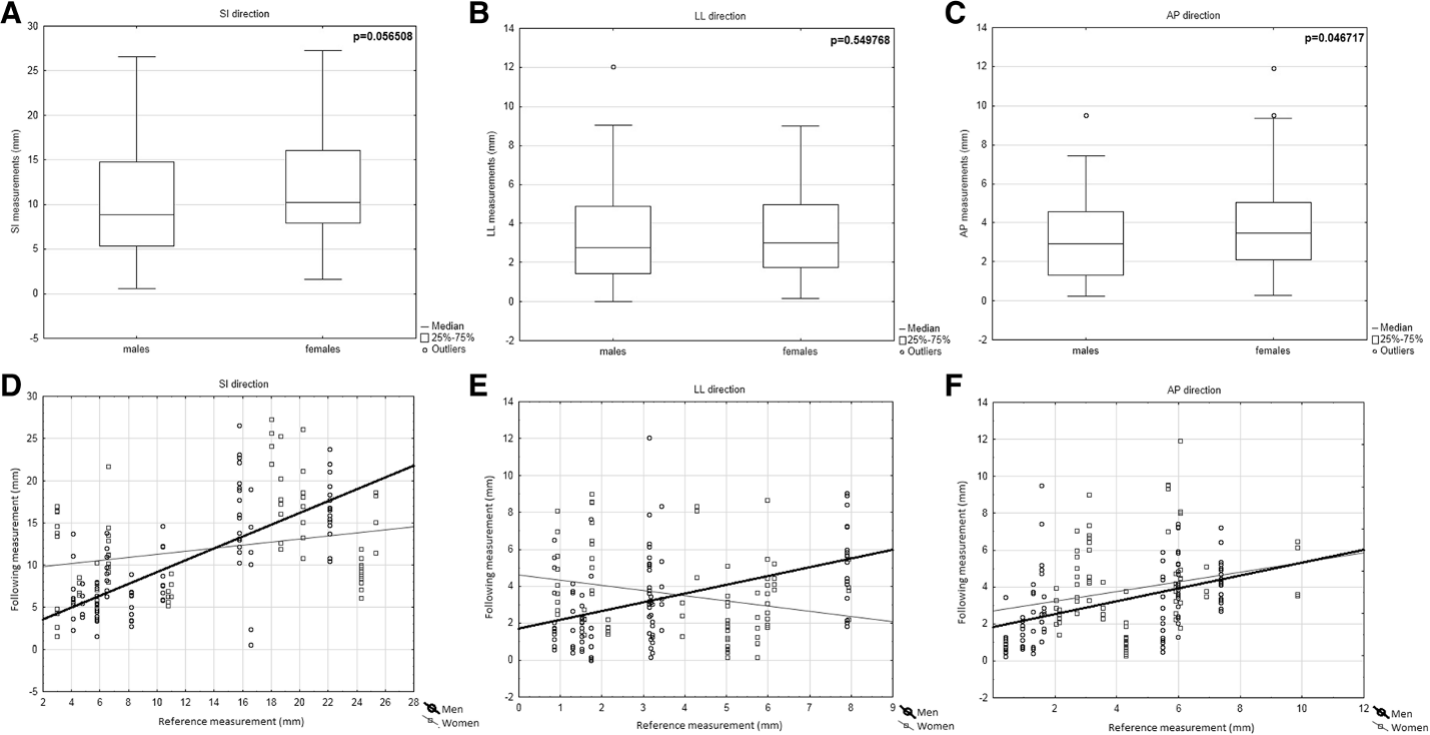
**Tumor motion amplitudes and variability of prediction for men and women.** We observed no significant difference between men and women regarding tumor motion amplitudes in all three directions: SI p = 0.056 **(A)**; LL p = 0.549 **(B)**; and AP p = 0.052 **(C)**. Variability of prediction for the SI direction **(D)**, men: y = 2.2008 + 0.7015*x; r = 0.7285; p = 0.0000; and women: y = 9.4583 + 0.1804*x; r = 0.2695; p = 0.0163. Test for difference between two correlation coefficients, p = 0.0000. Test for difference between two regression coefficients, p = 0.0001. LL direction **(E)**, men: y = 1.7543 + 0.4717*x; r = 0.4572; p = 0.000; and women: y = 4.6229-0.2843*x; r = -0.2505; p = 0.026. Test for difference between two correlation coefficients, p = 0.0000. Test for difference between two regression coefficients, p = 0.0001. AP direction **(F)**, men: y = 1.8117 + 0.346*x; r = 0.4538; p = 0.0000; and women: y = 2.7144 + 0.261*x; r = 0.1993; p = 0.0802. Test for difference between two correlation coefficients, p = 0.07. Test for difference between two regression coefficients, p = 0.57.

For the SI direction, we derived a “regression model” that added an extra margin to those acquired from the reference measurement (Figure 1A). As the results were far from optimal (only 2-3 mm margin reduction compared to a constant margin without reference measurement), we evaluated patient’s variability more closely. Women and men are known to favor different types of breathing (chest vs. abdominal). Our results did not indicate any significant difference regarding tumor motion amplitudes (Figure 2A-C) for all three directions, while the tumor motion prediction was higher for men, especially in the SI direction (Test for Difference Between Two Correlation Coefficients, p = 0.0000; Test for Difference Between Two Regression Coefficients, p = 0.0001) (Figure 2D; Table 4). Unfortunately, only modest margin reduction (1 mm) was seen.

### Correlation between internal and external markers during respiration

To get correlation error less than 3 mm (median 1.53 mm, range 0.01-3.09 mm), we had to establish more than 3 correlation models per treatment (median 9.5 times, range 4-18) whenever we observed a correlation error between internal and external markers greater than 3 mm. The median duration of one model was 19 min (range 1-87 min). 10 out of 20 patients (50%) were presented with low stability between internal and external markers and more than 9 models had to been done per treatment (3 fractions).

## Discussion

The treatment response of pancreatic tumors to radiotherapy treatment remains poor. Given the strong radioresistance of adenocarcinomas, dose escalation is needed but it is not possible without margin reduction [3–6].

In the present study, tumor motion was greatest in the SI direction, in agreement with data from other institutions [2, 9, 10, 13, 14]. According to our measurements (20 patients, monitoring time 4676 min), the mean respiration amplitude between inhalation and exhalation was 11 mm (range 5-23 mm), which appears to be comparable to previously published results from larger studies. Bussels et al. obtained their data by using dynamic MRI to quantify pancreatic motion in 12 patients; instead of placing fiducials, they acquired one image every second for 1 min. They observed a larger degree of movement in the SI direction: 24 mm ±16 mm [15, 16] compared to our result (1 minute of monitoring seems to be short and MRI tumor detection could be less precise). Although smaller tumor motion amplitudes have also been presented, those studies are based on a much smaller number of measurements [10, 13, 14]. Murphy et al. reported the results of only one patient imaged fluoroscopically for 1 min; the patient had three gold fiducials sutured into the tumor, and the maximal SI movement was 6 mm with breathing (13). Gierga et al. published a study of six patients who also underwent invasive marker placement and were observed fluoroscopically for 30 sec. The range of average SI motions was 4.4–12 mm (14). Hallman et al. concluded that the mean COM motion for pancreatic tumors was 5 mm (standard deviation 1 mm), with a range of 3 to 7 mm (10). We observed mean tumor motion of approximately 3 mm (range 3-7 mm) and 4 mm (range 3-8 mm) in the LL and AP directions respectively, which supports the recommendation for asymmetrical margins as published by Goldstein et al. [9].

To the best of our knowledge, this is the first study to quantify under- or overestimation of tumor motion during a longer time period. With the use of TAO and TAU, we were able to derive margins for the optimization of underdosage and overdosage. Our analysis clearly shows the feasibility of the concept of larger margin (20 mm) considered by the American-French consensus [7] for the SI direction, and lower (5 mm) for the LL and AP directions to account respiration. The use of smaller margins in the SI direction would cause an average underdosage zone of approximately 3 mm; moreover, 40% of patients would have an average geometrical miss of >3 mm. Abdominal compression might be useful [17] to minimize tumor motion, especially for the 50% of patients that presented with an average tumor motion of >15 mm. Limitations of our study could be using the tumor tracking system to simulate reference measurement instead of 4DCT and short time of monitoring (3 fractions in 1 week compared to 5 weeks of fractionated radiotherapy).

Planning 4D-CT is commonly used to customize the internal margin in patients with abdominal tumors [8]. Our results show that the motion of pancreatic tumors is modestly predictable with respiration in the SI direction (correlation coefficient r = 0.50) and unpredictable in the LL (r = 0.17) and AP (r = 0.35) directions when derived from the reference measurement. Margins based on the reference measurement of tumor motion avoid overdosage with an average geometrical miss of <3 mm in our group. However, 30% of patients have a risk zone of underdosage >3 mm caused by inter-/intrafractional changes of breathing pattern, that seems unacceptable. When Minn et al. [18] compared the planning of 4D-CT motion with intrafractional motion measured in a single-fraction respiratory tracking radiotherapy; they found that the geometrical miss could exceed 10% for 16 out of 20 patients. Compared with our results, they found an additional modest correlation for the AP direction. However, only the average value from all consequential measurement points was used, compared to our approach assessing the correlation between reference and subsequent measurements [18]. In a study of four pancreatic tumors monitored during 38 fractions, Ge et al. concluded that the motion measured with 4D-CT did not adequately represent actual motion during radiation therapy. The 4D-CT disagreed with 95% of the fractions, 55% of which were underestimated and 40% of which were overestimated [19]. Cai et al. found that gated internal volume based on 4D-CT could underestimate tumor motion in respiratory-gated therapy, mainly because of breathing variability [20]. James et al. found that the instability of internal target volume varied from 46% to 127% [21].

Our results are not in compliance with recommendations for the use of 4D-CT [9, 10]. Goldstein et al. observed that the volumes and excursions were relatively unchanged during the treatment course, obviating the need for re-planning during treatment [9]. However, the monitoring time was short, only two measurements were taken for 50% of patients, and inter-/intrafractional variability may have remained hidden; the duration of few breaths could not represent the entire treatment time. Hallman et al. recommend using respiratory gating towards the end of exhalation, which might substantially reduce the range of motion. They found an average shift of 5 mm (range 3-7 mm) for SI direction [10]. Abdominal motion was determined by one respiratory cycle, which could be a limitation of this study.

We have derived two methods of ITV optimization based on the reference measurement. Our algorithms allow us to reduce the margin (approximately from 20 mm to 17 mm for women and from 20 mm to 16 mm for men) in the SI direction, compared with the use of constant margins without reference measurement. No additional risk of underdosage was found. Our preliminary results showed much better prediction capability for men than for women, resulting in a smaller ITV and lower risk of overdosage for men. The amplitude analysis has not shown any significant difference between men and women (Figure 2A-C). Even after detailed analysis, the predictability of movement in both the LL and AP directions remains poor. Moreover, we have detected occasional unpredictable COM shifts caused by intestinal and duodenal peristalsis; therefore, another extra margin might be added.

Finally, we had to create approximately 200 correlation models with median duration of 19 min (range 1-87 min) to obtain good correlation between tumor motion and skin markers (error smaller than 3 mm). This finding is in agreement with the results of Feng et al., who demonstrated poor correlation between the tumor position and the abdominal wall and diaphragm. Poor reproducibility in breathing pattern has also been shown [16]. These additional uncertainties should be accounted for when image guidance with external markers (without fiducials) is used.

## Conclusion

Our results support the use of a 20-mm margin in the SI direction and a 5-mm margin in the LL and AP directions to account respiratory motion without a reference measurement. ITV optimization, based on the reference measurement, allows a small margin reduction in the SI direction (approximately from 20 mm to 16-17 mm) and no margin reduction in the LL/AP directions, with no additional risk of underdosage when compared with the use of constant margins. Further margin reduction is only possible when there is on-line tumor motion control according to internal markers.
